# Diagnostic Value and Clinical Application of Diffusion-Weighted Magnetic Resonance Imaging for Female Pelvic Lesions

**DOI:** 10.1155/2022/5868453

**Published:** 2022-06-20

**Authors:** Lulu Gao, Qing Lu, Yanting Wu

**Affiliations:** Affiliated Maternity and Child Health Care Hospital of Nantong University, Nantong, Jiangsu 226000, China

## Abstract

Pelvic inflammatory disease refers to a group of infectious diseases of the female upper genital tract, often caused by ascending infection of vaginitis and cervicitis, causing endometritis, salpingitis, tubo-ovarian abscess, pelvic connective tissue inflammation, and/or pelvic peritonitis. PID is the most common and important infectious disease in nonpregnant women of childbearing age, and inflammation in multiple parts often coexists and affects each other. The functional MRI techniques currently used in pelvic floor muscle injury are magnetic resonance diffusion tensor imaging, T2 mapping, and magnetic resonance elastography. Diffusion tensor imaging is a new imaging and postprocessing technology developed on the basis of magnetic resonance diffusion-weighted imaging. Due to the lack of specificity of clinical symptoms, many subclinical patients are often not detected and diagnosed in time, so it is very difficult to accurately estimate the incidence of PID. This article retrospectively analyzed 72 patients with pelvic inflammatory disease confirmed by surgical pathology from February 2020 to 2022, who had undergone pelvic MRI examination before surgery, including 25 patients with chronic pelvic inflammation (hydrosalpinx), 25 patients with acute pelvic inflammation, and 47 cases (including 21 cases of hydrosalpinx, 19 cases of tubo-ovarian abscess, and 7 cases of pelvic abscess). The age range was 13 to 59 years old. The clinical data and MRI findings were analyzed, the ADC value of the cystic part of the lesion was measured, and the differences in age, maximum diameter of the lesion, thickness of the vessel wall/separation, and the ADC value of the cystic part of chronic and acute pelvic inflammation were compared. In this part of the cases, there were 25 cases of chronic pelvic inflammation and 47 cases of acute pelvic inflammation. The average ADC value of the cystic component of chronic inflammation was significantly higher than that of acute inflammation, which were (2.86 ± 0.20) × 10^−3^ mm^2^/s and (1.07 ± 0.38) ×10^−3^ mm^2^/s, respectively, *P* value <0.001.

## 1. Introduction

Pelvic floor dysfunction (PFD) is a general term for pelvic floor structural and functional disorders, including pelvic organ prolapse, urinary incontinence, defecation incontinence, and perineal descent syndrome [1]. About 50% of women over the age of 50 in European and American countries are affected. Relevant epidemiological surveys in various regions of China have found that the incidence in females is about 22% to 30%, and the proportion of patients increases with age, parity, and weight [2–5]. The pathogenesis has not yet been elucidated. Since 1907, Fothergill proposed that ligaments play a major role in supporting the pelvic floor structure. In 1990, Petros proposed the “holistic theory.” The most widely recognized ones are the “Hammock Hypothesis” and “Three Vagina” proposed by DeLancey in 1992 and “Horizontal Support Theory” [6]. These theories are not only the basis for understanding the development and diagnosis and treatment of PFD, but also the foundation for the continuous development of pelvic floor imaging. Since Yang performed MRI scans on patients with pelvic floor dysfunction for the first time in 1991, with the development and updating of MRI machines and imaging techniques, pelvic floor MRI has gradually developed from static MRI, dynamic MRI, and functional MRI [7]. Combining a variety of MRI imaging techniques can not only help diagnose and analyze the changes of the three-chamber pelvic floor in real time, but also discover the changes and damage of anatomical structures under different pathological conditions and explore the pathogenesis [8].

Static MRI of the pelvic floor is scanned in a 1.5 T or higher field strength MRI device, and the recommended sequence is fast spin echo with fast acquisition of relaxation-enhanced sequences. Collecting high-resolution T2-weighted (T2WI) images of the pelvic floor in sagittal, coronal, and axial directions is helpful to observe changes in the anatomical shape and structure of the pelvic floor. Record the patient's complete history of pelvic floor disease, drink 300 mL of water after urination 2 hours before the examination, keep the bladder moderately full, and correctly train the patient's Valsalva action and anal retraction action. Provide urine pads during examination to increase their compliance [9]. The structures that need to be observed from the anterior, middle, and posterior pelvic support structures include urethral supporting ligament, puborectalis muscle; uterine cardinal ligament and sacral ligament, iliococcygeal muscle; pelvic diaphragmatic hiatus, rectovaginal septum, perineal body, etc. [10]. Recommended sequences include half-Fourier acquisition single-shot fast spin echo sequences, true steady-state precession fast-imaging sequences, and single-shot fast spin echo sequences. When posterior pelvic diseases are suspected (such as constipation due to outlet obstruction, rectocele, intestinal hernia, rectal intussusception, and prolapse), dynamic MR defecation angiography should be added, and 100–180 mL of ultrasonic coupling agent should be injected into the rectum [11–14]. In comparison of pelvic anatomy, the mid-sagittal, sagittal, coronal, and axial dynamic MRI images were collected every time the patient's resting phase-levator phase-relaxation phase-force-reflex phase was taken as a cycle [15]. The female pelvic floor has multiple layers of complex anatomical structures, and the muscles of the pelvic floor are like a “slinging net,” which tightly hangs the pelvic floor organs and maintains their normal position and function [16]. When the elasticity of the pelvic floor muscles becomes poor, the internal organs of the pelvic floor cannot maintain their normal positions, and the corresponding functions are impaired [17]. It is the only way to display the trajectory of muscle fiber bundles in vivo through the principle of anisotropy in the diffusion movement of water molecules in tissues [18] because the microstructure of tissue specimens will inevitably change during the processing process, resulting in geometric deformation. DTI adopts the cross-sectional spin echo-echo planar imaging (SE-EPI) technology, in which there are many parameters, and the most used are the diffusion sensitivity coefficient (b), the eigenvalues (*λ*_1_, *λ*_2_, *λ*_3_), average dispersion rate, fractional anisotropy, and apparent diffusion coefficient [19]. Among them, FA and ADC reflect the direction and amplitude of molecular diffusion motion, respectively. When the muscle is damaged, the structural order of the muscle fiber is destroyed, the diffusion of water molecules in all directions increases, and the FA value of the damaged muscle decreases [20, 21].

Based on the texture analysis method, this study carried out individualized analysis and prediction of the occurrence of pubic visceral muscle injury in pregnant women. To provide new diagnostic ideas and methods for pregnant women with PFD to help clinical diagnosis and treatment, in this study, Mann–Whitney *U* test and univariate logistic regression were used to screen out 351 texture features with statistically significant differences between normal fertile women's pubic visceral muscles and PFD patients' injured pubic visceral muscles, and then the *m* RMR feature selection was finally simplified to 7. A potential texture feature predicts pubic visceral muscle injury. At the same time, the clinical risk factors will have an impact on PFD: age, number of deliveries, and mode of delivery were subjected to univariate logistic regression. Finally, the age with statistical differences in this study was left as the clinical feature, which was jointly constructed with the texture feature prediction model. In texture features, wavelet has the best diagnostic performance (AUC = 0.87), and age has better diagnostic performance in clinical features (AUC = 0.84). Compared with the two methods for independently predicting pubic visceral muscle injury, the combined predictor had higher diagnostic performance (AUC = 0.96), indicating that the combined predictive model could better reflect potential pubic visceral muscle injury than traditional clinical features. MR-T2WI texture analysis method can more objectively extract texture features for analysis than traditional radiologists' observation of images for judging pubic visceral muscle injury and be combined with clinical independent risk factors to improve the accuracy of diagnosing pubic visceral muscle injury. MR-T2WI texture analysis method can quantitatively evaluate pubic visceral muscle damage in pelvic floor dysfunction, making the clinical efficacy evaluation more accurate and objective.

This article retrospectively analyzed 72 patients with pelvic inflammatory disease confirmed by surgical pathology from February 2020 to 2022, who had undergone pelvic MRI examination before surgery, including 25 patients with chronic pelvic inflammation (hydrosalpinx), 25 patients with acute pelvic inflammation, and47 cases (including 21 cases of hydrosalpinx, 19 cases of tubo-ovarian abscess, and 7 cases of pelvic abscess).

## 2. Methods

### 2.1. Research Objects

This study retrospectively collected and selected 54 pelvic floor dysfunction pregnant women admitted to our hospital from 2020 to February 2022 as the observation group, including 46 POP patients and 8 UI patients, aged 36–78 years old; The age was (59.0 ± 10.5) years old, the number of deliveries was 1 to 6 times, 52 cases were delivered by natural delivery, and 2 cases were delivered by cesarean section. At the same time, 40 pregnant women with normal pelvic floor function were selected as the control group, aged 28 to 64 years old, the number of deliveries 1 to 4 times, 32 cases of natural delivery, and 8 cases of cesarean section shown in Table 1. Urinary incontinence was based on the diagnostic criteria of the International Continence Association.

Inclusion and exclusion criteria of the observation group are as follows: (1) women who have given birth; (2) patients with pelvic organ prolapse clinically diagnosed according to POP-Q staging or urinary incontinence according to the diagnostic criteria of the International Continence Association; (3) no pelvic malignancy tumor disease. Inclusion and exclusion criteria for the control group are as follows: (1) women who have given birth; (2) no (symptomatic) pelvic floor disease; (3) no history of pelvic surgery; (4) no history of pelvic malignancy. When the lymph nodes are invaded by tumor cells and cause volume changes, MRI can evaluate the nature of enlarged lymph nodes by the short diameter of the lymph nodes. On MRI images, metastatic lymph nodes are mostly uniformly hypointense on T1-weighted images and hyperintense on T2-weighted images, while on fat-suppressed imaging, by fat-suppressing, metastatic lymph nodes are more prominent and volume can be displayed.

### 2.2. Instruments and Methods

A Siemens Avanto 1.5 T MR scanner was used with body phased array coils. The patient was placed in the supine position, and the bladder was moderately filled 2 hours before the scan. The scanning sequence and parameters selected in this study are horizontal axis T2WI Turbo Spin Echo (TSE) sequence, TR 2000–6581 ms, TE 72–129 ms, FOV 280 mm × 280 mm, matrix 320 × 320, averages 2.0, layer thickness 4.0 mm. The above scanning sequences and parameters are consistent with the recommended sequences and parameters for MRI examination of pelvic floor dysfunction recommended by the European Society of Urogenital Radiology and the European Society of Gastrointestinal and Abdominal Radiology Pelvic Floor Working Group in 2016.

**Figure 1:**
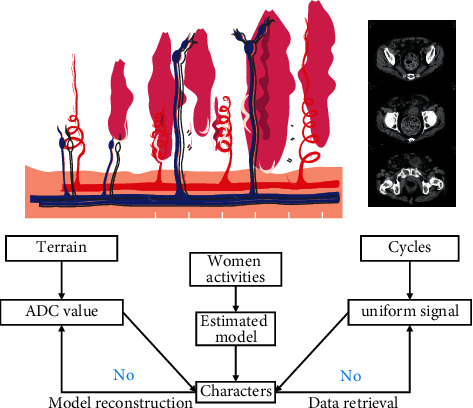
Magnetic resonance diffusion imaging setup.

The original images of the T2WI-FSE sequences of the observation group and the control group were exported and stored in DICOM format. The stored images were imported into ITK-SNAP software, and 2 radiologists with more than 5 years of experience in pelvic floor MRI diagnosis work in a blind way to delineate the region of interest (ROI). The ROI was delineated layer by layer along the contour of the pubic visceral muscle and then fused to obtain a global three-dimensional region of interest (3D-ROI) of the pubic visceral muscle (as shown in Figure 1). Save the exported ROI format as NII. Then, AK software (Analyze Kit, GE Healthcare, Shanghai) was used for feature extraction, including shape (shape), first-order histogram (first-order histogram), and gray-level size zone matrix (GLSZM), and change features include wavelet transform (WAV) features, Laplace variation features, 1219 features in total.

1. The intra- and inter-group correlation coefficients were used to evaluate the intra- and inter-measurer consistency, and the texture features with ICC > 0.75 were retained.
2. The differences in texture features between the observation group and the control group were compared using the Mann–Whitney *U* test, and the texture features with statistically significant differences between the groups were selected.
3. The second dimensionality reduction was performed by univariate logistic regression (*P* < 0.05).
4. Remove redundant and irrelevant features by using minimum redundancy maximum correlation feature selection.
5. Using the multivariate logistic regression model, the area under the curve corresponding to different characteristics and clinical risk factors was obtained, and the optimal AUC value was selected to construct the receiver operating characteristic line.
6. Linearly combine the selected features with their corresponding regression coefficients and clinical risk factors to calculate the imaging score of each patient. The higher the score, the greater its association with pubic visceral muscle damage.
7. Finally, cross-validation is used to prevent overfitting of the joint prediction model.

All statistical analyses were performed using R software 3.5.1, and *P* < 0.05 was considered statistically significant. Receiver operating characteristic curve (ROC) was used to evaluate the predictive value of texture features, and the sensitivity, specificity, and model accuracy. SPSS 25.0 statistical software was used to process the clinical characteristics of patients with pelvic floor dysfunction and normal pelvic floor function. Measurement data were analyzed by independent sample *t*-test or Wilcoxon rank sum test. Enumeration data were expressed by the number of cases, and comparisons were performed using the *χ*^2^ test or Fisher's exact test. *P* < 0.05 was considered statistically significant.

### 2.3. MRI Scanning Method

**Figure 2:**
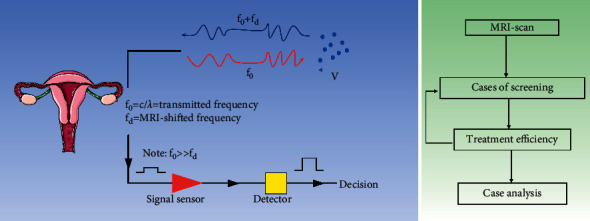
Lymphoid tissue imaging and construction.

All groups underwent routine abdominal and pelvic MRI scans and DWI scans, and all MRI scans were performed within one week before surgery. Before the examination, the patient was instructed to drink water to properly fill the bladder. After resting for 15 to 30 minutes, a routine pelvic MR sequence plain scan was firstly performed, including axial FSE T1WI (TR600 ms, TE7.4 ms, FOV 36 cm × 36 cm, matrix 224 × 320, acquisition times 2, slice thickness 6 mm, slice interval 1 mm), axial FSE T2WI (TR3820 ms, TE87.5 ms, FOV 36 cm × 36 cm, matrix 224 × 320, acquisition times 4, slice thickness 6 mm, slice interval 1 mm), and sagittal FSE T2WI (TR3100 ms, TE87.5 ms, FOV 30 cm × 30 cm, matrix 288 × 192, acquisition times 4, slice thickness 6 mm, slice interval 1 mm). The scanning range is from the inferior border of the pubic symphysis to the level of the bifurcation of the abdominal aorta. All MR images were subjected to postprocessing analysis of images and measurement of parameter values in a blinded manner by two experienced MRI diagnosticians (8 and 10 years of experience, respectively). The ADC value of the lymph node itself has a large heterogeneity. This study did not include patients with cervical adenocarcinoma, and for an enlarged lymph node with uniform signal, the region of interest when measuring the ADC value and eADC value should include 4/5 of the entire lymph node. If the signal of the enlarged lymph node is not uniform, the high signal area and low signal area of this lymph node are measured separately, as shown in Figure 2.

## 3. Analysis of Results

### 3.1. MRI Features of the Primary Tumor

**Figure 3:**
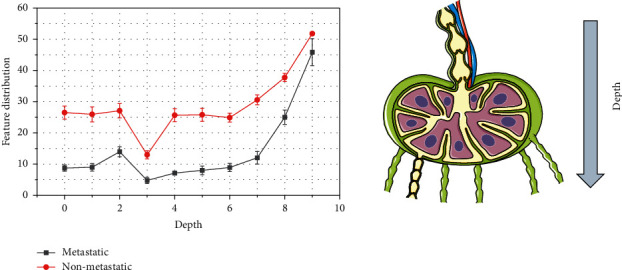
MRI feature distribution of the primary tumor.

Cervical cancer manifests as cervical thickening or volume enlargement on conventional MRI images. The primary tumor is often an irregular nodule or mass. It is iso- or slightly hypointense on T1WI-FSE images and slightly higher on T2WI-FSE images. Signal on DWI images (*b* value = 0 and 800 mm^2^/s) islow or slightly low signal on the corresponding ADC map, while the enhancement on enhanced T1WI image is uneven, and the enhancement degree is usually lower than that of normal muscle layer. Among the 61 patients with cervical cancer, 16 had cervical cancer primary tumor ≥4 cm and 45 had cervical cancer less than 4 cm measured on MRI images; the infiltration depth of the primary tumor was shallow 1/2. There were 41 cases of interstitial infiltration and 20 cases of deep 1/2 interstitial infiltration, as shown in Figure 3. At present, the treatment of cervical cancer is mainly based on extensive total hysterectomy + pelvic lymph node dissection and radical chemoradiotherapy, mainly based on cervical cancer staging.

### 3.2. Histopathological Findings of Lymph Nodes

According to the histopathological results of surgically resected specimens, a total of 1369 pelvic lymph nodes were surgically removed in this study. Combined with preoperative MRI images, a total of 127 pelvic lymph nodes that met the requirement of short diameter of ≥5 mm were identified (70 lymph nodes), accounting for 9.28% of the total number of surgically removed pelvic lymph nodes. The pathological types of 57 metastatic lymph nodes were squamous cell carcinoma or adenosquamous carcinoma, of which 52 (91.23%) were metastatic squamous cell carcinoma and 5 (8.77%) were adenosquamous carcinoma. All 70 nonmetastatic lymph nodes were pathologically reactive follicular hyperplastic lymph nodes. Most nonmetastatic lymph nodes are homogeneously isointense on T1WI images, and homogeneously or slightly hyperintense on T2WI images, and often show mild homogeneous enhancement on enhanced scans. In this group of cases, there was no central necrotic area and extracapsular invasion of nonmetastatic lymph nodes.

**Figure 4:**
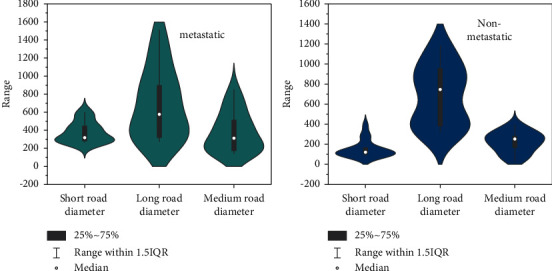
Comparison of the diameter difference between metastatic and nonmetastatic lymph nodes in the pelvis.

The specific distribution location and metastasis rate of metastatic lymph nodes in the pelvis are as follows: the first station includes the parametrial group and obturator group (1.17% and 14.68%); the second station includes the internal iliac and external groups and the deep inguinal group (13.12% and 14.68%, 10.41%); the third station included the general iliac group and the para-aortic group (8.03% and 1.22%). The diameter measurement results of pelvic lymph nodes are shown in Figure 4. Among the 57 metastatic lymph nodes, there were 31 lymph nodes with a short diameter of 5–10 mm, 22 lymph nodes with a short diameter of 10–15 mm, and 4 lymph nodes with a short diameter greater than 15 mm pieces. Among the 70 nonmetastatic lymph nodes, there were 53 lymph nodes with a short diameter of 5–10 mm, 17 lymph nodes with a short diameter of 10–15 mm, and 0 nonmetastatic lymph nodes with a short diameter greater than 15 mm. Statistical analysis showed that the long diameters of pelvic metastatic lymph nodes and nonmetastatic lymph nodes in cervical cancer patients were 14.2 ± 5.5 mm and 10.7 ± 3.9 mm, respectively, and the short diameters were 11.7 ± 3.3 mm and 6.8 ± 2.5 mm, respectively. The long-diameter ratios were 0.87 ± 0.21 and 0.61 ± 0.17, respectively, and there were significant statistical differences in the long-diameter, short-diameter, and short-diameter/long-diameter ratios between the two groups of pelvic lymph nodes (all *P* < 0.05).

The results of this study showed that pelvic metastatic lymph nodes of cervical cancer were often round or oval nodules on conventional MRI images, of which 68.4% (39/57) were round and 31.6% (18/57) were oval; most of the metastatic lymph nodes showed iso- or slightly hypointense signal on T1WI images, mainly high signal on T2WI images, some showed heterogeneous mixed high signal, enhanced scan showed obvious enhancement, and some necrosis without obvious enhancement was seen. Among them, 37 were uniformly enhanced, 20 were nonuniformly enhanced, and 35.1% (20/57) had necrotic areas with annular enhancement signs. There were 43 lymph nodes with clear borders and 14 with rough or blurred borders. Extracapsular invasion was seen in 33.3% (19/57) of the lymph nodes. On the other hand, most of the nonmetastatic lymph nodes were oval in conventional MRI images, and some of them were round, of which 62.9% (44/70) were oval or broad bean, and only 37.1% (26/70) were circular.

### 3.3. DWI Image Characteristics of Pelvic Lymph Nodes

**Figure 5:**
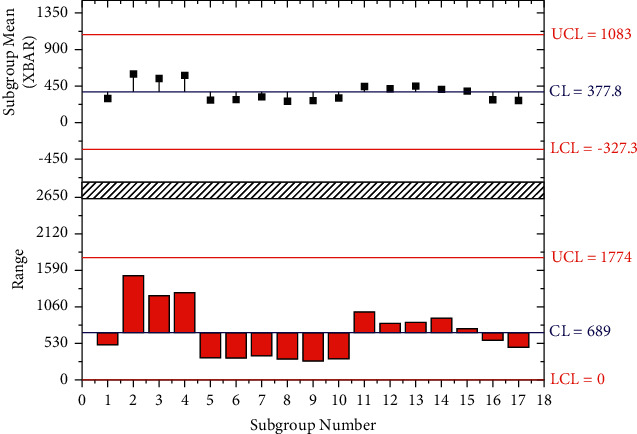
eADC values of metastatic lymph nodes in the pelvis.

The results of statistical analysis showed that the average ADC value and maximum ADC value of pelvic metastatic lymph nodes in patients with cervical cancer were significantly lower than those of nonmetastatic lymph nodes, the difference between the two was statistically significant (*t* = 3.359, *P*=0.003.; *t* = 5.661, *P* < 0.001), the average eADC value and minimum eADC value were also significantly higher than those of nonmetastatic lymph nodes, the difference between the two was also statistically significant (*t* = −5.156, *P* < 0.001; *t* = −4.594, *P* < 0.001); and there was no significant difference between the minimum ADC value and maximum eADC value of pelvic lymph nodes between the two groups (*t* = 1.722, *P* < 0.095; *t* = −0.907, *P*=0.216); see Figure 5 for details. The signal intensity of the lesion was divided into three types: high signal, low signal, and mixed signal according to the signal of muscle on DWI and ADC images with *b* values of 0 and 800 mm^2^/s. In order to eliminate the measurement error of ADC value and eADC value due to too small ROI, only lymph nodes with a short diameter of ≥0.5 cm were included in this study.

## 4. Discussion

### 4.1. Importance of Lymph Node Metastasis in Cervical Cancer

**Figure 6:**
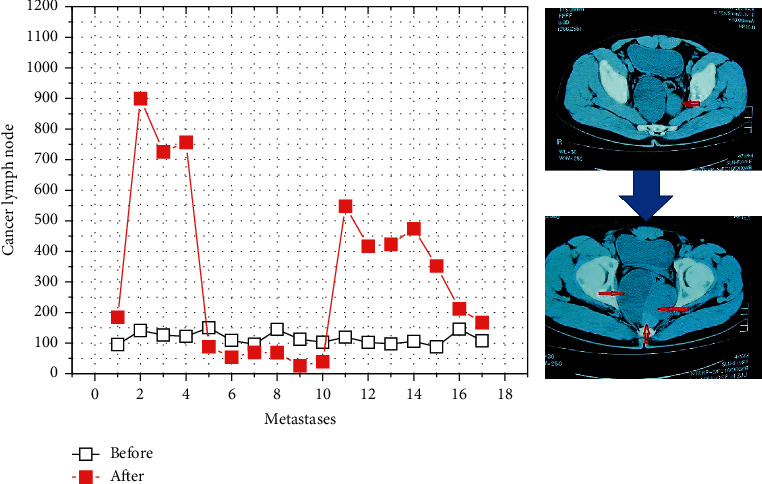
Survival changes before and after cervical cancer lymph node metastasis.

After surgical treatment of early cervical cancer patients, if the pathology suggests lymph node metastasis, the 5-year survival rate drops sharply from 85%–90% to 50%–55%, as shown in Figure 6. The lymph node metastasis rate of cervical cancer is related to its clinical stage. The lymph node metastasis rate of patients with stage I cervical cancer is 15–20%, that of stage II is 25–40%, and that of stage III patients can be as high as more than 50%. It can be seen that the higher the stage, the greater the possibility of pelvic lymph node metastasis. In addition, the number of lymph node metastases is a prognostic factor for cervical cancer recurrence and disease-free survival. For patients with stage IB-IIA cervical cancer, the 5-year survival rate was similar with one lymph node metastasis and no lymph node metastasis, but the 5-year survival rate decreased if the number of metastatic lymph nodes was more than one. When there are more than 4 lymph node metastases, the prognosis is worse. For patients with advanced cervical cancer in stage IIB and above, radiotherapy is the main treatment, and surgery cannot bring additional survival benefits, but it may delay the timing of radiotherapy and lead to complications related to surgery; for patients with lymph node metastasis, postoperative treatment is required. Therefore, if the pelvic lymph node metastasis can be accurately assessed before treatment, it can provide important information and guidance for clinical decision-making and avoid the dual treatment of surgery and radiotherapy.

### 4.2. Lymph Node Metastasis and Histological Types of Cervical Cancer

**Figure 7:**
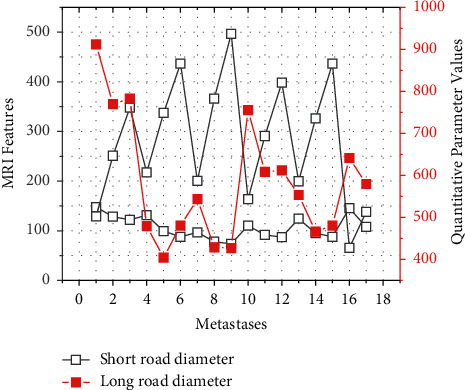
MRI features and quantitative parameter values to identify pelvic metastases.

Compared with cervical squamous cell carcinoma, cervical adenosquamous carcinoma is more aggressive and has a worse prognosis. It has been reported that the lymph node metastasis rates of cervical adenosquamous carcinoma and cervical squamous carcinoma patients with the same clinical stage and tumor size are 21% and 15%, respectively, and the distant metastasis rate (DMR) is 37% and 37%, respectively. 21% suggests that cervical cancer patients with pathological type adenosquamous carcinoma are more prone to lymph node metastasis. In recent years, the incidence of cervical adenosquamous carcinoma has gradually increased. The data show that in cervical adenosquamous carcinoma and cervical squamous cell carcinoma receiving postoperative radiotherapy or concurrent chemoradiotherapy (CCRT), the overall survival and disease-free stage of cervical adenosquamous carcinoma are significantly worse than those of cervical squamous cell carcinoma. The reason may be related to factors such as the late discovery period of cervical adenosquamous carcinoma, the lower sensitivity to radiotherapy than cervical squamous carcinoma, and the easier metastasis of adenosquamous carcinoma, as shown in Figure 7. However, the number of cervical adenosquamous carcinomas included in this study is small, which may lead to bias in data analysis, and the results need to be verified by a large-sample prospective study. In this study, it was found that MRI assessed the presence or absence of pelvic lymph node metastasis by measuring the short diameter, morphology, maximum ADC value, average ADC value, and average eADC value of lymph nodes, and the differences were statistically significant. It can be seen that MRI has important diagnostic value for judging cervical cancer pelvic lymph node metastasis.

The previous FIGO clinical staging of cervical cancer depends on the clinical examination of the gynecological oncologist. Regardless of the imaging results, the initial clinical staging does not change. The new 2018 FIGO guidelines include pelvic and para-aortic lymph node metastases into staging, allowing imaging findings to modify the initial clinical staging. The FIGO guidelines do not specify which imaging assessment method should be used. When combined with local inflammation, reactive lymph node enlargement and tumor metastatic lymph node enlargement are often difficult to distinguish on imaging images, so it is necessary to be cautious in evaluating lymph node metastasis by imaging examination. The selection of imaging examination methods with high accuracy has important guiding significance for clinical decision-making. At present, the commonly used imaging methods for preoperative diagnosis of cervical cancer lymph node metastasis mainly include ultrasound, CT, MRI, and PET/CT. Ultrasound examination has its unique advantages, mainly in that it is easy to operate, nonradioactive, reproducible, and inexpensive. It is a common examination method for various gynecological diseases. The direction of the probe can be changed arbitrarily during the ultrasound examination, so the suspicious lesions can be observed comprehensively, and the vaginal ultrasound probe can observe the lesions at close range, which is a unique advantage of ultrasound compared with other examinations.

**Figure 8:**
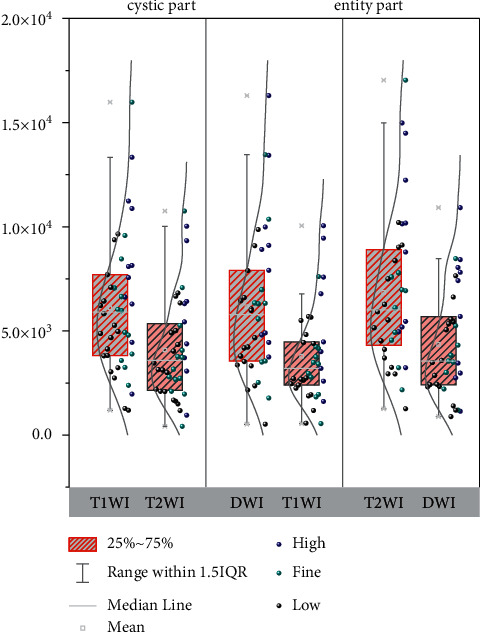
Comparison of diagnostic signals of cervical cancer lymph node metastasis.

Ultrasound has strong diagnostic value in the diagnosis of cervical cancer lymph node metastasis. The main image characteristics are as follows: the volume of lymph nodes increases, and the length-to-width ratio of metastatic lymph nodes is mostly >0.5; Upper metastatic lymph nodes are characterized by abundant blood flow signals displayed by color Doppler ultrasound, as shown in Figure 8.

## 5. Strengths and Limitations

Magnetic resonance imaging (MRI) is a tomography technique. By applying radio frequency pulses of a specific frequency to tissue cells in a static magnetic field, hydrogen protons are excited to produce magnetic resonance. The process is called relaxation, and protons generate MR signals during the relaxation process. MRI is a noninvasive examination method that does not generate ionizing radiation and can perform multi-sequence and multi-directional imaging, making it more and more widely used in clinical practice. MRI has high resolution for soft tissue, and multi-section scanning can clearly display the structures of the cervix, uterus, vagina, parametrium, and adjacent organs; degrees are higher.

The main sequences of MRI to evaluate cervical cancer include T1-weighted image (T1WI) and T2-weighted image (T2WI). T1WI can clearly show the pelvic anatomy, and T2WI has advantages in judging the boundary between cancer foci and normal tissue. Retrospective analysis of the MRI data and pathological results of 67 patients with cervical cancer showed that the diagnostic accuracy of MRI for invasive cervical cancer was 95%, and the diagnostic accuracy for staging was 76%. A multicenter study conducted suggested that MRI is more accurate than CT in diagnosing lymph node metastases in cervical cancer. With the development of technology, diffusion-weighted imaging (DWI) detects the diffusion movement of water molecules in tissues from the molecular level, which indirectly reflects the abnormality of tissue and cell structure and function and metabolism, which is a supplement to conventional MRI examinations. The apparent diffusion coefficient (ADC) value measured by DWI can quantitatively analyze the diffusion degree of water molecules and improve the accuracy of diagnosis. Therefore, the combination of DWI, ADC values, and conventional MRI images can improve the diagnostic value of MRI in identifying cervical cancer and evaluating whether cervical cancer has lymph node metastasis.

With the development of science and technology, the application of contrast-enhanced ultrasound has greatly improved the resolution of ultrasonography and its ability to display microvessels, which can fully demonstrate the blood perfusion of lesions and make up for the shortcomings of conventional ultrasound. However, ultrasonography is still easily interfered by the abdominal wall fat layer, intestinal gas signal in the abdominal cavity, bladder, etc., which may easily lead to missed diagnosis of lesions and suspicious lymph nodes; the ultrasonic detection range is small, and it is difficult to make a comprehensive assessment of the entire pelvic situation; the resolution of ultrasonography is relatively high. The anatomical relationship between the lesion and surrounding tissues and organs cannot be well displayed, and it is difficult to detect lymph nodes with small diameters.

## 6. Future Research

When the lymph nodes are invaded by tumor cells and cause volume changes, MRI can evaluate the nature of enlarged lymph nodes by the short diameter of the lymph nodes. On MRI images, metastatic lymph nodes are mostly uniformly hypointense on T1-weighted images and hyperintense on T2-weighted images, while on fat-suppressed imaging, by fat-suppressing, metastatic lymph nodes are more prominent and volume can be displayed. . At present, most studies use lymph node short diameter of >1 cm as the diagnostic criterion to judge lymph node metastasis. A meta-analysis of 42 literature on MRI diagnosis of cervical cancer lymph node metastasis by Liu et al. showed that the overall sensitivity of MRI was 54% and the specificity was 93%. When the short diameter of >1 cm was used as the criterion for positive lymph node diagnosis of cervical cancer, the accuracy of MRI could reach 87%. The results of our study showed that among the pelvic metastatic lymph nodes, there were 31 lymph nodes with 0.5 cm ≤short diameter <1 cm, 22 lymph nodes with 1 cm ≤short diameter <1.5 cm, and 4 lymph nodes with 1.5 cm ≤short diameter. Among the lymph nodes, 53 were 0.5 cm ≤short diameter <1 cm, 17 were 1 cm ≤short diameter <1.5 cm, and 0 were 1.5 cm ≤short diameter. Statistical analysis showed that the short diameters of pelvic metastatic lymph nodes and reactive hyperplastic lymph nodes in patients with cervical cancer were 11.7 ± 3.3 mm and 6.8 ± 2.5 mm, respectively, and the short diameters of pelvic lymph nodes between the two groups were significantly different (*P* < 0.05).). However, the data show that metastatic lymph nodes are mostly small in size, and 54% (31/57) of metastatic lymph nodes with a short diameter of <1 cm. Therefore, if only the size of the lymph nodes is used to determine whether the pelvic lymph nodes have metastasized, it is easy to cause a low sensitivity and a missed diagnosis rate higher. In the future, if the pelvic lymph node metastasis can be accurately assessed before treatment, it can provide important information and guidance for clinical decision-making and avoid the dual treatment of surgery and radiotherapy.

## 7. Conclusion

This article retrospectively analyzed 72 patients with pelvic inflammatory disease confirmed by surgery and pathology from February 2020 to 2022 who had undergone pelvic MRI examination before surgery, including 25 patients with chronic pelvic inflammation (hydrosalpinx) and 47 patients with acute pelvic inflammation (including 21 cases of hydrosalpinx, 19 cases of tubo-ovarian abscess, and 7 cases of pelvic abscess). The age range was 13 to 59 years old, with an average age of 41.3 ± 10.5 years. The clinical data and MRI findings were analyzed, the ADC value of the cystic part of the lesion was measured, and the differences in age, maximum diameter of the lesion, thickness of the vessel wall/separation, and the ADC value of the cystic part of chronic and acute pelvic inflammation were compared. In this part of the cases, there were 25 cases of chronic pelvic inflammation and 47 cases of acute pelvic inflammation. There was no significant difference in mean age and maximum diameter between the chronic inflammation group and the acute inflammation group (*P* > 0.05). The thickness of the vessel wall/partition in the acute inflammation group was significantly greater than that in the chronic inflammation group (*P* < 0.05), 11.48 ± 6.47 mm and 2.53 ± 1.04 mm, respectively. The average ADC value of the cystic component of chronic inflammation was significantly higher than that of acute inflammation, the two were (2.86 ± 0.20) × 10^−3^ mm^2^/s and (1.07 ± 0.38) × 10^−3^ mm^2^/s, *P* value<0.001. The mean ADC values of pyosalpinx, tubo-ovarian abscess, and pelvic abscess in acute inflammation were (1.13 ± 0.26) × 10^−3 ^ mm^2^/s, (1.05 ± 0.45) × 10^−3^ mm^2^/s, and (1.09 ± 0.36) × 10^−3 ^ mm^2^/s; there was no statistical difference among the three groups. Magnetic resonance imaging (MRI) is a tomography technique.

## Figures and Tables

**Table 1:** Basic information of trainees.

Research group	Number of people	Sub-group	Number of people	Age
Observation group	54	POP patients	4 6	59.0 ± 10.5
		UI patients	8	

Control group	4 0	POP patients	3 2	62.45 ± 6.45
		UI patients	8	

## Data Availability

The data used to support the findings of this study are included within the article.
